# Zirconium-Based Metal–Organic Framework Nanomaterials for Antibacterial Applications: Nanoscale Design and Structure–Function Relationships

**DOI:** 10.3390/nano16201314

**Published:** 2026-10-18

**Authors:** Yanxiang Kang, Di Zhao, Xinran Fang, Yunwei Niu

**Affiliations:** Faculty of Flavour Fragrance and Cosmetics, Shanghai Institute of Technology, No. 100 Haiquan Road, Shanghai 201418, China; kyx@sit.edu.cn (Y.K.); zhaodi@sit.edu.cn (D.Z.); embrace1117@163.com (X.F.)

**Keywords:** zirconium-based metal–organic frameworks, antibacterial nanomaterials, antibacterial applications, controlled release delivery, functional nanocomposites

## Abstract

Bacterial contamination and biofilm-associated infections require antimicrobial materials that combine effective bacterial control with tunable transport, structural stability, and acceptable safety. Zirconium-based metal–organic frameworks (Zr-MOFs) are attractive platforms because their Zr-oxo clusters, pore environments, surface chemistry, and defect structures can be adjusted over multiple length scales. Rather than treating synthesis, antibacterial mechanisms, and applications as separate topics, this review organizes current evidence around a nanoscale design–structure–function framework. We examine how particle size, defect density, pore accessibility, surface chemistry, and hybridization influence bacterial contact, guest loading and release, reactive oxygen species (ROS) generation, photothermal conversion, and biofilm-related performance. Antibacterial systems are distinguished according to the contribution of the Zr-MOF host, incorporated active species, and externally triggered processes, and mechanistic claims are considered according to the strength of the reported experimental evidence. Representative applications in wound care, antibacterial textiles, food packaging, water disinfection, and electrochemical systems are discussed together with the experimental conditions and safety constraints that limit direct cross-study comparison. Particular attention is given to the trade-offs between increased accessibility or reactivity and framework stability, leaching, and cytotoxicity. This structure–function perspective is intended to provide practical design guidance for developing more reproducible, interpretable, and application-relevant Zr-MOF antibacterial nanomaterials.

## 1. Introduction

Infectious diseases have long been recognized as a major threat to global human health and a severe burden on public health systems [1,2]. Bacterial infections rank as the second-leading cause of death worldwide, threatening millions of lives [3]. Over the past decades, antibiotics, agents capable of inhibiting or eliminating bacteria, have played a major role in curbing the spread of bacterial infections. However, the overuse and misuse of conventional antibiotics have accelerated the development of antimicrobial resistance, which the World Health Organization lists among the gravest public-health risks worldwide [4,5,6]. To more effectively combat infections caused by bacteria, fungi, and other microorganisms, researchers have extensively investigated novel, high-performance antibacterial agents. A wide range of antibacterial substances is currently in use. These include inorganic additives (silver (Ag) [7], copper (Cu) [8], zinc (Zn) [9], and their compounds, alongside photocatalysts such as titanium dioxide (TiO2) [10], tin dioxide (SnO2) [11], and zinc oxide (ZnO) [12]; organic antibacterial agents (including organic halides [13], acids [14], and ketones [15]); and naturally derived active ingredients including berberine [16], curcumin [17], and chitosan [18]. However, these materials still have several limitations, including insufficient bactericidal activity, inadequate structural stability, and intrinsic biotoxicity [19,20]. Consequently, there remains a need to develop novel antimicrobial agents. Metal–organic frameworks (MOFs) are porous materials with periodic network structures self-assembled from metal ion clusters and organic building units via coordination bonds [21]. Compared with conventional zeolites and other inorganic porous materials, MOFs exhibit substantial structural diversity. Different metal ions, organic ligands, and assembly modes give rise to diverse structures and physicochemical properties [22]. Owing to their high specific surface areas, readily tunable pore geometry, abundant coordinatively unsaturated metal sites, and tunable chemical compositions, MOFs are widely used in catalysis [23], molecular separation [24], gas storage [25], water and air pollutant remediation [26], and electrochemistry [27]. Moreover, their low cytotoxicity and favorable biodegradability support broad applications in biomedical research [28,29]. Their flexible crystal lattices and high porosity also facilitate the loading and encapsulation of antibacterial agents and bacteria-targeting functional moieties. MOFs also offer several advantageous properties, including facile surface modification, the availability of antibacterial metal ions, abundant redox-active sites, and excellent enzyme-mimicking catalytic activity. These characteristics make MOF-based materials important functional platforms in antibacterial research [30].

Several major classes of MOF-based antibacterial materials are summarized below. (a) ZIF series. Zeolitic Imidazolate Frameworks (ZIFs) are self-assembled via tetrahedral coordination between Zn^2+^/Co^2+^ cations and imidazole-based organic linkers [31]. Combining the advantages of conventional MOFs and natural zeolites, ZIF-type frameworks offer favorable specific surface areas, pore volumes, and structural stability [32]. Previous studies have shown that Zeolitic Imidazolate Framework-8 (ZIF-8), Zeolitic Imidazolate Framework-67 (ZIF-67), Zeolitic Imidazolate Framework-L (ZIF-L), and other ZIF derivatives exhibit strong bactericidal activity [33,34,35]. (b) Materials of the Institute Lavoisier (MIL) series. MIL stands for Materials of the Institute Lavoisier. These porous hybrid frameworks are synthesized from trivalent transition-metal ions and dicarboxylate linkers such as glutaric acid and can be divided into two major groups. Early MIL materials were prepared from lanthanide species or transition-metal oxides coordinated with organic diprotic-acid linkers, including succinic acid and organophosphoric acid, to construct the framework [36]. More recent MIL compounds generally use terephthalate-type carboxylate linkers coordinated with trivalent metal ions. Well-known representatives, Materials of the Institute Lavoisier-53 (MIL-53), Materials of the Institute Lavoisier-101 (MIL-101), and Materials of the Institute Lavoisier-125 (MIL-125) [37,38] have all been reported to exhibit antibacterial activity [39,40,41]. (c) IRMOF series. Isoreticular Metal–Organic Frameworks (IRMOFs) were first fabricated by Yaghi and his research team [42]. Researchers adjusted linker length and functionalized terephthalate linkers before coordinating them with octahedral Zn_4_O^6+^ clusters. This approach yielded a series of isoreticular MOFs with tunable pore sizes. In addition to these MOF families widely investigated for antibacterial applications, Zr-MOFs have also attracted substantial research interest.

Constructed from zirconium-containing inorganic polyatomic clusters cross-linked by multidentate organic linkers, Zr-MOFs constitute an important class of metal–organic porous materials. In 2008, Karl Petter Lillerud’s group at the University of Oslo first reported a zirconium-centered framework named University of Oslo-66 (UiO-66), with the molecular formula Zr_6_O_4_(OH)_4_(BDC)_6_ and terephthalic acid (H_2_BDC) as the organic linker [43]. The high coordination number of Zr(IV) together with strong Zr-O bonds stabilizes the framework. These bonding characteristics give UiO-66 excellent resistance to harsh aqueous and chemical environments, as well as favorable mechanical stability under 1.0 MPa and thermal stability up to 500 °C [44]. Derived from the UiO-66 prototype by extending the organic linkers, University of Oslo-67 (UiO-67) and University of Oslo-68 (UiO-68) share the same network topology. The University of Oslo (UiO) family and its various hybrid derivatives have attracted extensive interest in antibacterial research [45,46]. Carboxylic-acid porphyrin-based Zr-MOFs constitute another important class of zirconium-based frameworks, self-assembled through coordination bonds between zirconium ion clusters and organic porphyrin or metalloporphyrin ligands [47]. Among carboxyl-porphyrin Zr-MOFs, porphyrin linkers bind to zirconium metal clusters in three common coordination modes with coordination numbers of 6, 8, and 12. Representative examples include Porous Coordination Network-223 (PCN-223) (12-coordinated) [48], Porous Coordination Network-222 (PCN-222) (8-coordinated) [49], and Porous Coordination Network-224 (PCN-224) (6-coordinated) [50]. The Porous Coordination Network (PCN) series was first reported by Hongcai-Zhou’s research team. This class of highly stable porous MOFs is constructed with tetravalent zirconium ions as inorganic coordination nodes and porphyrin units as organic linkers [51,52,53]. The PCN family contains numerous subtypes. Early studies mainly focused on gas adsorption and storage, while subsequent studies have extended their applications to drug-delivery systems [54] and antibacterial food-packaging materials [55]. Numerous other representative Zr-MOFs, including Northwestern University-1000 (NU-1000) and Metal–Organic Framework-808 (MOF-808), also show promising potential for antibacterial applications.

**Figure 1:**
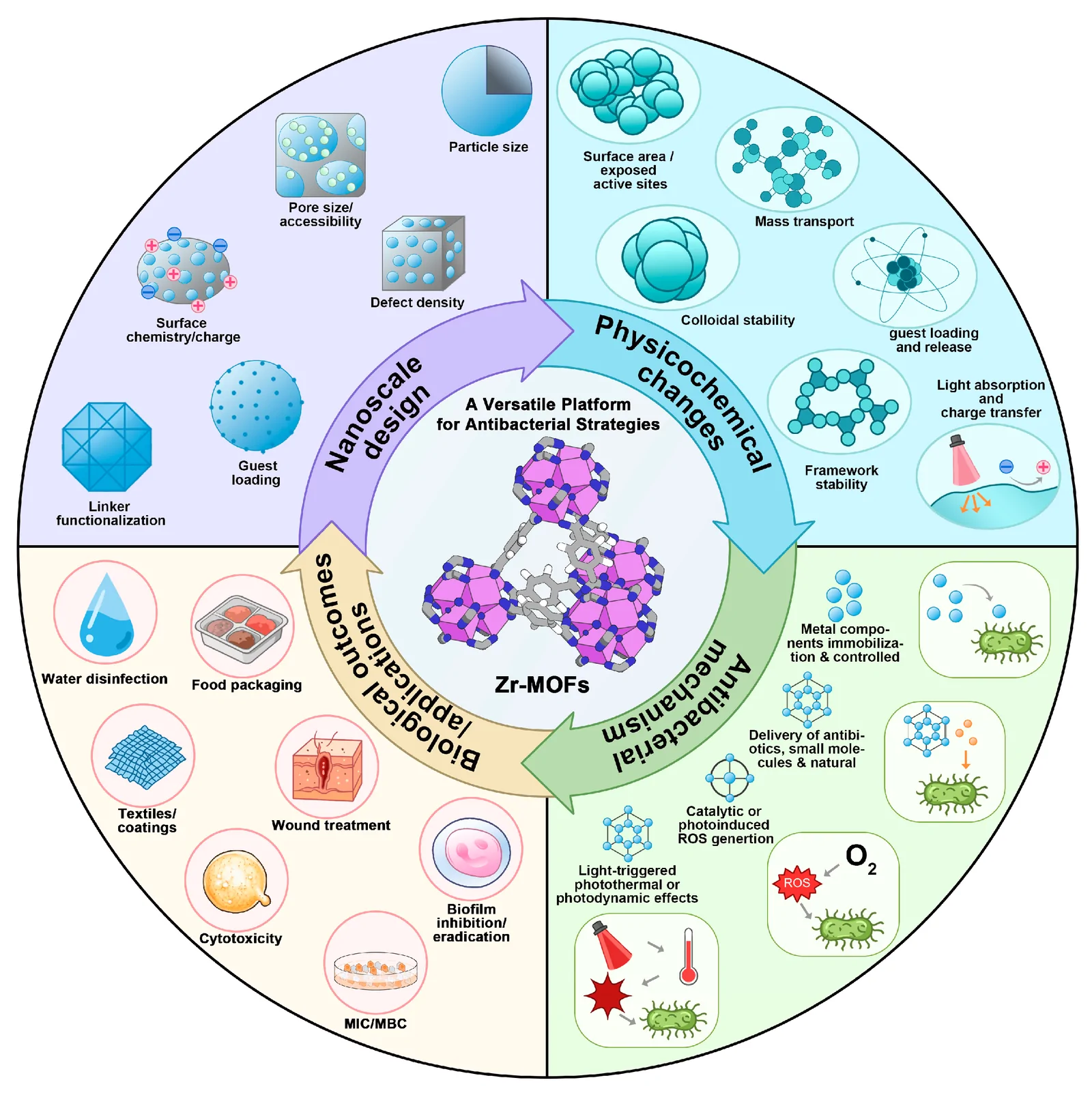
Schematic illustration of Zr-MOFs nanomaterials for antibacterial applications.

Recent reviews have established important foundations for Zr-MOF synthesis and biomedical use [56], UiO-based antibacterial, anti-inflammatory, and anticancer platforms [57], and UiO-based drug-delivery systems with antimicrobial and other applications [58]. These studies provide broad coverage of synthesis routes, therapeutic functions, and representative applications. The present review is differentiated by using nanoscale design variables as the organizing framework and by asking how measurable structural features translate into physicochemical consequences, antibacterial mechanisms, and biological outcomes. Specifically, we focus on particle size, defect chemistry, pore accessibility, surface chemistry/charge, and hybrid architecture; distinguish intrinsic Zr-MOF effects from the activity of loaded metals, drugs, natural products, or photoactive components; and emphasize the comparability and limitations of reported antibacterial data. This design-variable-to-outcome framework is then applied across wound care, textiles, food packaging, water disinfection, and electrochemical antibacterial systems to identify both transferable design principles and application-specific trade-offs (Figure 1).

This article is a critical narrative review rather than a systematic review. For the present revision, the evidence base was refined by cross-comparing the recent Zr-MOF/UiO reviews cited above [56,57,58] and tracing representative primary studies relevant to antibacterial structure–function relationships. Priority was given to studies reporting a defined Zr-MOF composition together with at least one material descriptor (for example, particle size, porosity, defect state, surface chemistry, or payload characteristics) and a measurable antibacterial or antibiofilm endpoint. Studies centered only on sensing, pollutant adsorption/degradation, pesticide delivery, antifungal/antiviral activity, or non-antibacterial therapeutic effects were excluded from quantitative antibacterial comparisons unless they directly informed a material-design principle. Because the underlying literature is highly heterogeneous, comparisons are interpreted with attention to strain, dose, exposure time, irradiation conditions, test format, and the presence or absence of Zr-MOF-only controls.

Literature selection in this narrative review follows an evidence-focused strategy instead of PRISMA systematic review guidelines. Primary antibacterial studies were prioritized if they reported microbial performance together with material structural characterization or matched control groups. The literature search was performed using Web of Science, with the following search terms: ‘zirconium-based metal–organic frameworks”, “antibacterial nanomaterials”, “antibacterial applications”, “controlled release delivery”, and “functional nanocomposites”, covering the past decade. These search parameters were documented during literature screening rather than reconstructed from the reference list.

## 2. Nanoscale Design of Zr-MOFs for Antibacterial Function

Zr-MOFs can be produced through solvothermal, mechanochemical, microwave-assisted, electrochemical, and sonochemical routes [59,60,61,62]. For antibacterial materials, however, the synthetic route is relevant only insofar as it controls properties that affect biological performance. Reaction temperature, precursor concentration, modulator chemistry, solvent environment, energy input, and reaction time can alter nucleation and growth, particle-size distribution, morphology, defect population, pore accessibility, and surface functionality. These parameters in turn influence colloidal behavior, bacterial contact, active-species loading and release, mass transport, and framework stability. Accordingly, this section compares the major synthesis routes not only by operational features but also by the structural attributes that they can tune and the trade-offs that may emerge in antibacterial applications.

### 2.1. Solvothermal Synthesis

Solvothermal synthesis is one of the most widely used routes for Zr-MOF fabrication, but it should not be treated as synonymous with a one-pot method. In a solvothermal process, metal precursors and organic ligands react in a solvent at elevated temperature, typically in a sealed vessel; a one-pot process instead describes the number and sequence of reaction steps and may be implemented under different heating modes. For UiO-type Zr-MOFs, nucleation and crystal growth are highly sensitive to pH, precursor concentration, temperature, reaction time, water content, and modulators [30,63]. Changing these conditions can alter crystallite size and defect formation as well as the accessibility of Zr sites and pores. Thus, the antibacterial relevance of solvothermal synthesis lies less in the route itself than in the resulting particle size, surface chemistry, pore environment, and defect population. Conventional UiO-66 is commonly obtained from zirconium precursors and terephthalic acid in DMF followed by heating and post-synthetic washing [64], while UiO-67, PCN-222, and PCN-224 can also be prepared by related solvothermal protocols [65,66,67]. The route offers high crystallinity and broad compositional flexibility, but long reaction times, solvent use, batch-to-batch sensitivity, and scale-up in pressurized vessels remain practical limitations.

### 2.2. Mechanical Grinding Synthesis

Mechanochemical synthesis uses mechanical energy to drive framework formation with little or no bulk solvent [68,69,70]. Compared with conventional solvothermal synthesis, shorter processing times and reduced solvent demand can improve sustainability and may facilitate scale-up [71]. Importantly, mechanical treatment can also change crystallite size, defect concentration, and accessible surface sites rather than simply reproducing a solvothermally prepared phase. High-porosity NU- and UiO-type frameworks have been prepared by mechanochemical routes [72], illustrating that rapid synthesis can retain useful porosity. For antibacterial design, these structural consequences are potentially advantageous because smaller crystallites and more accessible sites may improve contact or loading; however, excessive disorder, amorphous impurities, or uncontrolled defect formation can reduce framework stability and reproducibility. Mechanochemical synthesis should therefore be evaluated through both production metrics and the resulting nanoscale structure.

### 2.3. Microwave-Assisted Synthesis

Microwave-assisted synthesis provides rapid volumetric heating and can accelerate nucleation and crystal growth [73,74,75]. Microwave power, solvent dielectric properties, precursor concentration, water/modulator content, and reaction time can substantially change particle size, crystallinity, and defect population. Continuous tubular microwave reactors have enabled rapid UiO-66 production [76], while greener solvent systems have been explored for UiO-66 and MOF-808 [77]. Microwave processing can therefore be useful when small particles or rapid, reproducible nucleation are desired, but the antibacterial implications depend on whether the resulting changes improve pore accessibility, surface exposure, guest loading, or photoactivity without compromising structural stability. Scale-up, heat/mass transfer uniformity, solvent safety, and protocol standardization remain important constraints.

Microwave irradiation can produce MOF phases that are difficult to obtain by conventional thermal synthesis. Nevertheless, volatile organic solvents contained in sealed reaction vessels and nonstandard operating procedures may create explosion hazards, imposing inherent safety limitations on this synthetic strategy.

### 2.4. Electrochemical Synthesis

Electrochemical synthesis forms MOFs under an applied electrical potential and can proceed through electrode-surface nucleation, anodic dissolution, cathodic deposition, or related electrochemical routes [78,79,80]. This method offers short reaction times and tunable electrochemical parameters, and UiO-67 and UiO-66-NH2 have been prepared under mild conditions [81,82]. From a structure–function perspective, current density, potential, precursor transport, and deposition time can influence nucleation density, particle morphology, film thickness, and interfacial adhesion. These variables are particularly relevant when the Zr-MOF is intended as a coating or electrode-integrated antibacterial material. The principal challenge is reproducibility: small variations in electrochemical conditions can change crystal quality and surface coverage, which can in turn alter redox behavior, mass transport, and antibacterial performance.

### 2.5. Sonochemical Synthesis

Sonochemical synthesis uses acoustic cavitation to create transient high-temperature and high-pressure microenvironments that promote rapid nucleation [83,84]. It can shorten synthesis times and favor small crystallites or mesoporous structures; porphyrinic Zr-MOFs and mixed UiO/MIL phases have been produced by this route [85,86]. Template-free sonochemical UiO-66 with nanoscale grain size has also been reported [87]. For antibacterial systems, reduced particle size and increased pore accessibility may improve bacterial contact and active-species transport, but these advantages should not be assumed to translate directly into higher antibacterial efficacy. Intense sonication can introduce disorder, mixed phases, or framework damage, and these changes may compromise hydrolytic stability or batch reproducibility. Sonochemical products should therefore be compared using both structural metrics and application-relevant antibacterial endpoints.

### 2.6. Cross-Cutting Nanoscale Design Variables

Across these synthesis routes, particle size is a recurring design variable because it changes accessible surface area, dispersion, sedimentation/colloidal behavior, and the frequency of contact with bacterial envelopes. Smaller particles can shorten diffusion distances and may improve penetration into heterogeneous biofilm structures, but they can also increase nonspecific exposure to mammalian cells. Accordingly, a simple “smaller is better” rule is not supported; particle size should be interpreted together with surface charge, aggregation state, dose, and the biological test format [30,88,89].

Defect engineering provides a second cross-cutting variable. Missing-linker and missing-cluster defects can create coordinatively unsaturated Zr sites, alter pore connectivity, and increase transport or guest-accessible volume, but excessive defects may weaken hydrolytic or mechanical stability. The functional consequence therefore depends on defect type, density, spatial distribution, and the species used to cap or compensate the defective sites [88]. For antibacterial materials, these variables can influence adsorption and release of active guests, access of substrates to catalytic sites, and the stability of the framework during aqueous exposure. Defect metrics should therefore be reported quantitatively where possible rather than inferred only from synthesis conditions.

Pore accessibility and surface chemistry connect these structural features to the biological interface. Larger or better-connected pores can facilitate loading and transport of antibacterial guests, whereas surface functional groups and charge influence dispersion, protein adsorption, and interactions with bacterial envelopes. These variables are coupled: functionalization can reduce accessible pore volume, guest loading can change surface charge, and defect creation can simultaneously increase open sites and leaching susceptibility. Antibacterial design should therefore be treated as a multivariable optimization problem rather than as independent maximization of surface area, porosity, or defect density [20,30,88,89,90].

Importantly, a correlation between particle size, BET area, or missing-linker content and catalytic transport does not by itself demonstrate an antibacterial structure–function relationship. Direct evidence requires controlled Zr-MOF material series with comparable bacterial assays, whereas extrapolations from general defect engineering or pollutant degradation should be labeled as design hypotheses.

## 3. Nanoscale and Interfacial Antibacterial Mechanisms

The antibacterial performance of Zr-MOF-based materials arises from an interplay between nanoscale structure and the identity of the active component. Particle size and surface charge influence dispersion and bacterial contact; pore accessibility and defect sites regulate loading, transport, and release; and linker/guest chemistry determines whether light-driven, catalytic, or drug-delivery pathways are available. These relationships are not necessarily monotonic. Smaller particles can increase accessible surface area and contact with bacterial envelopes but may also increase nonspecific cellular exposure, while additional defects can create open sites and faster transport at the expense of framework stability [88]. For this reason, the following discussion separates intrinsic or host-mediated effects from the activity of loaded species and distinguishes mechanisms supported by direct experimental evidence from those inferred primarily from material composition or antibacterial outcome [30,89].

### 3.1. Antibacterial Mechanism via Metal Ion Release from Zr-MOF Hosts

Antibacterial metal ions such as Cu^2+^ [91], Ag^+^ [92], and Co^2+^ [93] can damage bacterial cells through membrane interactions, protein/enzyme binding, redox imbalance, and other intracellular effects. Their activity and toxicity depend strongly on ion identity, concentration, speciation, and exposure conditions. In Zr-MOF antibacterial composites, the Zr(IV) framework should not be conflated with these strongly bactericidal ions: free zirconium species generally show much weaker direct antibacterial activity, while the Zr-MOF more often functions as a stable scaffold, reservoir, or reaction platform. Incorporating Ag-, Cu-, or other active components can enable controlled release and improve dispersion, but the resulting antibacterial effect should be attributed to the composite only when appropriate Zr-MOF-only controls are available. Likewise, ROS production, membrane disruption, and nanozyme activity should be described as experimentally demonstrated mechanisms only when supported by direct assays; otherwise, they are better treated as proposed or inferred pathways.

**Figure 2:**
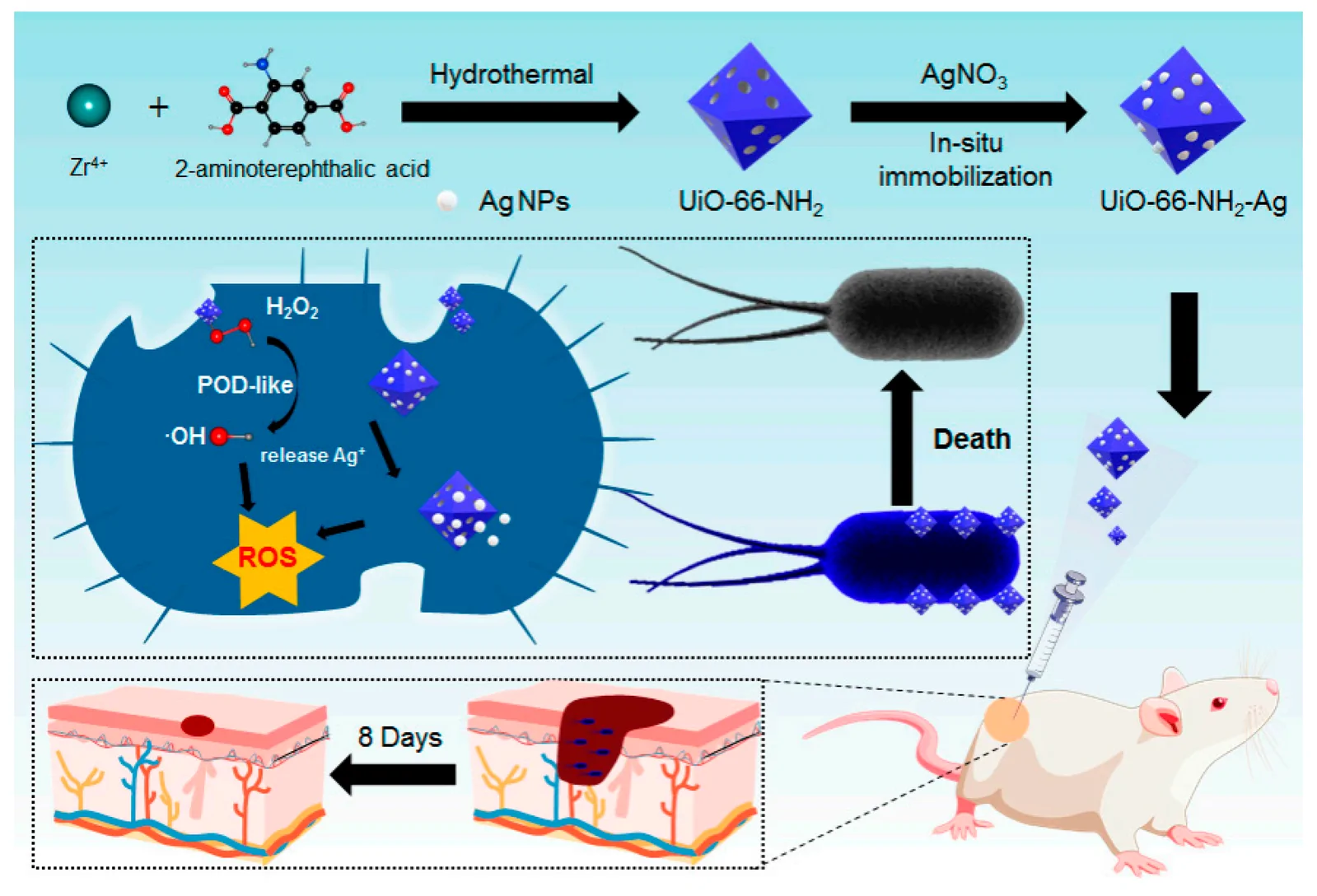
Schematic illustrations of the synthesis process of well-dispersed Ag NPs anchored with porous MOF-based UiO-66-NH_2_ to form UiO-66-NH_2_-Ag nanocomposite and their peroxidase-like activity and synergistic Ag^+^ release for highly efficient and rapid bacterial elimination. Reproduced from Ref. [94] under the terms of the CC BY 4.0 license.

Zhou and colleagues used an in situ immobilization approach to anchor silver nanoparticles (AgNPs) onto UiO-66-NH_2_. This procedure yielded the nanocomposite silver-loaded amino-modified University of Oslo-66 (UiO-66-NH_2_-Ag), namely, Zr-based 2-amino-1,4-benzenedicarboxylate loaded with silver nanoparticles. Characterization data show that silver nanoparticle functionalization imparts strong peroxidase-mimetic (POD-like) nanozyme activity to UiO-66-NH_2_-Ag, thereby accelerating the elimination of antibiotic-resistant bacterial strains. This composite exhibits effective bactericidal activity against ampicillin-resistant *E. coli* and spore-forming Bacillus subtilis, even at a low dosage of 12 μg/mL [94] (Figure 2). Gao’s team synthesized a silver-doped zirconium porphyrin MOF using the bottle-around-ship encapsulation strategy. This broad-spectrum antibacterial framework was covalently attached to cotton textiles pretreated with reactive tetrahydroxymethyl chitosan groups. After a series of laundering cycles, the fabric was subjected to mechanical abrasion, immersion in artificial sweat solutions, and prolonged storage under ambient conditions. The modified fabric retained antibacterial efficacy above 85% [95]. Chiericatti and his coworkers incorporated silver nanoparticles into UiO-66 to prepare antifungal nanocomposites. After a 72 h incubation period, the Ag/UiO-66 reduced the viable Penicillium roqueforti colony counts by five orders of magnitude, confirming strong antifungal activity [96].

### 3.2. Antibacterial Effects of Antiseptic Substances Loaded into Zr-MOFs

In recent years, Zr-MOFs have attracted substantial attention as porous carrier matrices. Many small organic molecules, including conventional antibiotics, exhibit bactericidal activity and are used to treat bacterial infections. However, these free molecules often have limited structural stability and can readily agglomerate or become deactivated in water, which markedly reduces their practical disinfection efficiency [97]. Owing to their rigid crystalline frameworks and interconnected pore networks, Zr-MOF hosts can stabilize these organic guest molecules and improve their overall antibacterial efficacy [98].

#### 3.2.1. Drug-Loading System

Zr-MOFs can serve as carriers for antibacterial drugs because their pore networks and surface chemistry permit high loading and sustained or stimulus-responsive release [99,100]. For example, UiO-66-NH2 has been used to load cefazolin, with antibacterial and antibiofilm testing supporting sustained activity and subsequent wound-healing studies [101]. Such systems illustrate a key distinction for structure–function analysis: the Zr-MOF may improve drug stability, localization, or release kinetics without being the dominant bactericidal component itself. Antibacterial performance should therefore be interpreted against controls containing the unloaded Zr-MOF and the free drug whenever these data are available. Studies centered primarily on non-antibacterial payloads or unrelated therapeutic endpoints are not considered strong evidence for Zr-MOF antibacterial function and are not emphasized here.

#### 3.2.2. Immobilization of Small-Molecule Antibacterial Substances

Beyond conventional antibiotics, Zr-MOF pores and surfaces can immobilize small-molecule antibacterial compounds and thereby modify their stability, local concentration, and release profile. A representative example is UiO-66-based encapsulation of caffeic acid, which achieved sustained release and was associated with damage to *E. coli* and *S. aureus* cell structures [22]. UiO-66-containing chitosan scaffolds have also been used as reservoirs for fosfomycin and showed antibacterial activity against *S. aureus* [102]. In evaluating such systems, release kinetics and loading capacity are useful material descriptors, but they should be connected to antibacterial dose, exposure time, strain, and appropriate carrier-only controls before a structure–function relationship is inferred.

#### 3.2.3. Immobilization of Plant-Derived Natural Products

Plant-derived compounds can provide antibacterial activity but often suffer from volatility, poor aqueous solubility, or difficult-to-control release. Zr-MOFs can mitigate some of these limitations by immobilizing or encapsulating the active compound. Rezaee and coworkers grew Zr-MOF layers on wool fibers and immobilized sage and calendula extracts; the resulting fabrics inhibited *E. coli* and showed partial inhibition of *S. aureus* [103]. For this class of materials, the antibacterial contribution of the plant-derived payload should be separated from any effect of the Zr-MOF carrier. Studies focused only on antioxidant, anti-inflammatory, anticancer, or other non-antibacterial endpoints are therefore outside the core evidence base of this review and are not used to support antibacterial structure–function claims.

### 3.3. Light-Triggered Antibacterial Activity

Light-triggered antibacterial strategies use photoinduced heat or reactive species to damage bacterial cells and can provide spatial and temporal control over treatment [104,105,106]. These approaches may reduce reliance on conventional antibiotics and can lessen some forms of antibiotic-selective pressure, but they should not be described as intrinsically incapable of inducing microbial adaptation. Their performance also depends on irradiation wavelength, power density, exposure time, oxygen availability, light penetration, and the properties of the surrounding medium. These experimental variables should therefore be considered together with antibacterial outcome when comparing photothermal and photodynamic Zr-MOF systems.

#### 3.3.1. Photothermal Therapy

Photothermal therapy (PTT) relies on photothermal agents that convert light energy into heat under laser irradiation. The localized heat generated by this process can inactivate bacteria within biofilms and disrupt the biofilm structure, thereby enabling broad-spectrum prevention and treatment of biofilm-associated infections [107,108]. Duan and coworkers constructed cellulose-paper-based composite films designated zirconium-based tetrakis(4-carboxyphenyl)porphyrin metal–organic framework@polydopamine (PDA)@fluorescent-protein (Zr-TCPP@PDA@FP), using polydopamine and zirconium-porphyrin MOF as the main components. Flexible membranes exhibited strong photothermal bactericidal activity, achieving inactivation rates above 99% for both *Escherichia coli* and *Staphylococcus aureus* [109]. Zhao’s research group grew cuprous-oxide nanoparticles on two-dimensional Zr-Fc-MOF nanosheets. The resulting 2D composite material enabled near-infrared (NIR)-assisted, photothermally enhanced chemodynamic antibacterial therapy. The photothermal effect under 808 nm near-infrared laser irradiation further enhanced the chemodynamic activity of the as-synthesized sample [110]. Wang and coworkers introduced carboxyl-terminated polyethylene glycol (PEG) (COOH-PEG-COOH) onto Zr-Fc-MOF and subsequently constructed a cross-linked hydrogel system designated PEG@Zr-Fc-MOF hydrogel. Its favorable photothermal properties enhanced catalytic activity and enabled synergistic antibacterial action. Wound-infection model tests verified that this hydrogel exhibited potent bactericidal activity, accelerated tissue regeneration, and showed negligible biological toxicity. These findings support the potential of combined photothermal-nanozyme-hydrogel platforms for bacterial wound-infection management [111]. Luo’s research group used a Prussian-blue (PB)-based MOF as the core to fabricate a porphyrin-doped PB@MOF heterostructure. This material achieved a photothermal conversion efficiency of 29.9% under 808 nm NIR radiation. After 10 min of dual-wavelength irradiation with 808 nm near-infrared light and 660 nm red light, the heterogeneous interface of PB@MOF achieved antibacterial rates of 99.31% against *Staphylococcus aureus* and 98.68% against *Escherichia coli* [112].

#### 3.3.2. Photodynamic Therapy

Photodynamic therapy (PDT) operates through the following mechanism. Upon photon absorption under laser irradiation, photosensitizer molecules transition from the ground state to an excited state. The absorbed energy is then transferred to oxygen molecules and surrounding substrates, generating highly reactive ROS [113,114]. Upon irradiation at an appropriate wavelength, ROS cause cellular damage and inhibit bacterial proliferation. They are also capable of inducing lethal damage in tumor cells and pathogenic microorganisms [115,116]. Chen and their research team synthesized pristine Porous Coordination Network-224 metal–organic framework (PCN-224 MOF) from zirconium tetrachloride and meso-tetrakis(4-carboxyphenyl)porphyrin (TCPP) linkers. Some of the original zirconium nodes were replaced with titanium ions to produce mixed-metal Porous Coordination Network-224 nanoparticles with zirconium–titanium bimetallic clusters (PCN-224 (Zr/Ti)). Experimental data confirmed enhanced photocatalytic activity of these hybrid particles. Abundant radical species were generated upon light exposure. The material showed strong antibacterial activity against multiple drug-resistant strains, including methicillin-resistant *Staphylococcus aureus*, methicillin-resistant Staphylococcus epidermidis, and drug-tolerant *Escherichia coli* [117]. Ahmad and coworkers embedded titanium-dioxide nanosheets into the UiO-67 framework and constructed polarized Zr-O-Ti active sites. A set of TiO_2_(X)@ZBPU composites was subsequently fabricated, where X denotes the titanium molar fraction (0.5, 1, and 2). Tests demonstrated that TiO_2_(2)@ZBPU exhibited markedly enhanced photocatalytic activity. Over the same reaction period, it achieved complete inactivation of *Escherichia coli* and over 90% removal efficiency for *Staphylococcus aureus* [118]. Chen’s research group anchored cobalt-doped amino-modified University of Oslo-66 nanoparticles based on zirconium-cobalt bimetallic clusters (UiO-66-NH_2_ (UiO(Zr/Co))) onto electrospun membranes composed of carboxylated cellulose nanocrystal (CCNC)-polyacrylonitrile (PAN) blended substrates. This produced a nanofibrous film. Under visible light illumination, the fiber membrane activated peroxymonosulfate (PMS) and removed viscous crude-oil contaminants while mitigating biological fouling, demonstrating strong photocatalytic self-cleaning performance and bactericidal activity [119]. Xu’s research team incorporated porphyrin compounds and ammonium glycyrrhizinate into MOFs to form dual-ligand Zr-MOFs. The synergistic antibacterial effect was achieved by combining the membrane-disrupting ability of porphyrin with the inhibitory effect of ammonium glycyrrhizinate on bacterial membrane synthesis, realizing a bactericidal rate of up to 99.999% [120].

### 3.4. Synergistic Effects of Multiple Antibacterial Pathways

Multimodal antibacterial systems combine pathways such as photothermal heating, photodynamic ROS production, catalytic ROS generation, metal ion release, or controlled drug delivery. Synergy can improve killing under conditions where a single pathway is insufficient, but a higher antibacterial rate alone does not establish mechanistic synergy. Strong mechanistic assignments require controls that isolate the individual components or stimuli and, where relevant, direct measurements of ion release, ROS, temperature, membrane integrity, enzyme-like activity, or drug release. Throughout this review, experimentally demonstrated pathways are therefore distinguished from mechanisms inferred mainly from material composition or endpoint antibacterial activity [30,89].

Evidence strength is considered at three levels: direct mechanistic evidence (mechanism-specific perturbation or controls linked to bacterial endpoints), indirect support (ROS probes, release profiles, or microscopy without a causal control), and proposed mechanisms (plausible but untested explanations). Antibacterial activity of Ag, Pt, Au, porphyrin, or antibiotic payloads must not automatically be assigned to the Zr-MOF host.

Xie and coworkers designed a photodynamic antibacterial composite comprising dithiol-modified University of Oslo-66 loaded with tetrakis(4-carboxyphenyl)porphyrin and silver nanoparticles (UiO-66-(SH)_2_@TCPP@AgNPs) through silver nanoparticle encapsulation and TCPP doping. Both the embedded silver nanoparticles and porphyrin-based TCPP enhanced visible light absorption and increased ROS production. Through the combined effects of silver encapsulation and porphyrin doping, the composite achieved strong antibacterial activity against *Escherichia coli*, *Staphylococcus aureus*, and methicillin-resistant *Staphylococcus aureus* (MRSA) strains [121]. Zhang’s research group coated hyaluronic acid (HA) onto a silver-ion-loaded photosensitive porphyrin MOF and constructed a surface-adaptive antibacterial nanoplatform designated silver-loaded Porous Coordination Network-224 modified with hyaluronic acid (PCN-224-Ag-HA) with synergistic antibacterial properties. When target bacteria were present, secreted hyaluronidase degraded the outer HA shell. Positively charged nanoparticles were exposed, releasing silver ions and generating ROS under visible light irradiation. The system therefore exhibited synergistic bactericidal activity [122].

Photothermal treatment acts synergistically with metal ion release to enhance bactericidal activity. Zhao and coworkers used a light-driven reduction strategy to grow silver nanoparticles on 2D MOF nanosheets and fabricate zirconium-ferrocene-based MOF sheets. Their photothermal properties accelerated Ag^+^ release and increased bacterial-membrane permeability. Silver nanoparticles released silver ions into the surrounding medium through oxidation. Under light irradiation, these ions penetrated bacterial envelopes more readily. Once inside bacterial cells, Ag^+^ induced endogenous ROS accumulation and oxidative stress, resulting in efficient synergistic disinfection [123]. Wu’s research group combined acid-activated chemodynamic therapy with mild photothermal treatment to design the injectable hydrogel dressing Zr/Fc-MOF@CuO_2_@FH. The Zr/Fc-MOF framework served as both a photothermal agent and a nanozyme for ROS generation. The outer cupric-peroxide layer decomposed in the acidic microenvironment of infected sites and released Cu^2+^ and hydrogen peroxide, which further boosted the catalytic activity of Zr/Fc-MOF. Both in vitro and in vivo experiments verified the strong bactericidal efficacy of this strategy against methicillin-resistant Staphylococcus aureus (MRSA) and kanamycin-resistant Escherichia coli (KREC), supporting its potential for clinical antibacterial treatment [124].

Four additional primary studies strengthen the comparison. Pt-NP/UiO-66 combines catalytic singlet-oxygen generation with biofilm-matrix disruption and macrophage responses [125]. Au nanocluster-modified porphyrinic PCN-224 has been evaluated for light-assisted killing and infected diabetic wound healing [126]. A controlled topology comparison of MOF-525, MOF-545, PCN-223, and PCN-224 links framework topology to singlet-oxygen generation and photodynamic antibacterial activity [127]. Benzoselenadiazole-doped Zr-MOFs provide a separate light-activated example with experimental biofilm inhibition [128]. These outcomes differ in assay design and cannot be compared as a common efficacy ranking.

To make cross-study comparison more transparent, representative Zr-MOF antibacterial systems are summarized below (Table 1). Only values explicitly reported in the cited studies are included; NR indicates that the corresponding parameter was not reported in the source used here. Because bacterial strains, doses, exposure formats, and irradiation conditions differ substantially, the reported antibacterial efficiencies should not be interpreted as directly interchangeable.

The reported results in Table 2 are not directly rankable across studies because bacterial strains, concentrations, exposure times, illumination, and assay endpoints differ. MIC/MBC values should not be inferred from percentage killing or log-reduction data. Host, payload, and external-stimulus contributions are distinguished wherever experimental controls permit. All available quantitative descriptors reported in the selected studies are provided without omission in Supplementary Table S1. The complete 15-column evidence matrix is provided as Supplementary Table S1; NR denotes information not reported by the original study, not a measured zero.

## 4. Antibacterial Applications of Zr-MOFs Nanocomposites

The practical value of a Zr-MOF antibacterial system depends on the application environment as much as on its maximum laboratory killing efficiency. Consequently, antibacterial results obtained with different strains, material doses, exposure times, irradiation conditions, suspension/surface formats, biofilm models, or in vivo systems should not be treated as directly interchangeable. This section therefore discusses representative applications in wound dressings, antibacterial textiles, food packaging, water disinfection, and electrochemical antibacterial systems using application-specific criteria. Studies centered on pesticide delivery, pollutant adsorption, sensing alone, or other functions without direct microbial inactivation are outside the core antibacterial scope and are not used as primary evidence for antibacterial performance.

### 4.1. Wound Dressings

The skin is both a physical and immune barrier, and bacterial colonization can markedly delay the repair of chronic or infected wounds [131,132]. Zr-MOF-containing dressings can integrate controlled release, catalytic ROS generation, photothermal or photodynamic action, and hydrogel/fibrous support functions [133,134]. However, wound-healing efficacy should not be inferred from antibacterial activity alone. Relevant evidence includes activity against planktonic bacteria and mature biofilms, cytocompatibility, hemocompatibility where appropriate, stability in protein- and exudate-rich media, inflammatory response, and in vivo tissue repair. Claims of “biocompatibility” or “low cytotoxicity” should therefore be restricted to the tested cell type, dose, exposure time, and formulation rather than generalized to the complete Zr-MOF platform [135,136,137].

Wu’s research team encapsulated quercetin with anti-inflammatory and antibacterial properties into amino-functionalized UiO-66 and subsequently loaded the nanoparticles into an aerogel constructed from agarose, aldehyde hyaluronic acid, and carboxymethyl chitosan to obtain ACA-MOF@Que, targeting different stages of wound healing. Experiments demonstrated that this polysaccharide-based aerogel possesses antioxidant, anti-inflammatory, and antibacterial properties [138]. Chen and coworkers used a photodynamic strategy to combine PCN-224 nanoparticles with titanium components, and then anchored the composite onto poly(lactic-co-glycolic acid) (PLGA) nanofibers to fabricate wound dressings. Experimental results showed that the dressing exhibited good biocompatibility and negligible cytotoxicity. It substantially accelerated in vivo healing in chronic wounds infected with multidrug-resistant bacteria [117]. Another research team led by Chen synthesized near-infrared-responsive composite hydrogel dressings by incorporating upconversion nanoparticles (UCNPs)@platinum-modified zirconium-based metal–organic framework (UCNPs@Zr-MOF-Pt) nanoparticles. Under near-infrared laser irradiation, the upconversion nanoparticles emitted visible light. The emitted light activated the porphyrin-based MOF dispersed within the hydrogel network and generated singlet oxygen (^1^O_2_) for photodynamic antibacterial treatment, thereby accelerating tissue repair [139].

Catalytic pathways can also act synergistically with photothermal effects within hydrogel matrices. Wang and coworkers modified zirconium-ferrocene MOF nanosheets with COOH-PEG-COOH and embedded the nanosheets in carrageenan-based hydrogel matrices. The as-prepared PEG@Zr-Fc-MOF hydrogel exhibited strong bactericidal activity and accelerated wound tissue regeneration. The material exhibited negligible cytotoxicity and reduced inflammatory responses, supporting its potential for bacterial wound-infection therapy [110]. Song’s research group reported a dibenzaldehyde-modified, polyethylene glycol cross-linked zirconium-based metal–organic cage/chitosan composite hydrogel. The zirconium-containing cages catalyzed the conversion of trace hydrogen peroxide into hydroxyl radicals. The composite outperformed conventional hydrogels in inhibiting *Escherichia coli* and *Staphylococcus aureus*. MTT assays together with live/dead cell characterization confirmed its favorable cytocompatibility. It also accelerated wound healing in infected-skin animal models [140].

### 4.2. Antibacterial Textiles

Porous and hydrophilic textile fibers provide a large accessible surface area. They tend to trap microbes, moisture, and nutrients, thereby promoting bacterial adhesion and proliferation. This issue is particularly important for protective garments, face masks, and medical-grade fabrics. Microbial contamination of these materials may compromise hygiene and user safety. Zr-MOFs possess tunable chemical compositions, pore microenvironments, and catalytic sites. Their rigid frameworks can be firmly immobilized as porous surface coatings, making them suitable candidates for the fabrication of high-performance antibacterial textiles.

Ma et al. incorporated persulfate, a stable peroxide precursor, into two porous Zr-MOFs. When exposed to humid air, hydrolysis triggered the sustained release of bactericidal hydrogen peroxide. These active precursors exhibited good storage stability and could be regenerated after exhaustion. These persulfate-loaded Zr-MOF frameworks were grown in situ on textile substrates by an aqueous method and exhibited reusable antibacterial performance against both Gram-negative and Gram-positive bacteria. This design provided humidity-responsive antibacterial activity without continuous external irradiation. However, the release rate and long-term peroxide retention require further evaluation under practical conditions, including variable humidity, repeated washing, and fabric wear. Photocatalytic disinfection provides another strategy [141]. Wang’s research group introduced titanium into the zirconium-pyrene-based Northwestern University-1000 metal–organic framework (NU-1000 MOF) and synthesized highly reactive bactericidal photocatalysts, which were subsequently coated onto cotton fabrics. Under simulated sunlight, the composite material achieved a 7-log reduction (99.99999%) within one hour against Gram-negative bacteria, Gram-positive bacteria, and T7 bacteriophage. These studies demonstrate that bimetallic Zr-MOFs are promising self-sterilizing coating candidates. However, their practical utility depends on photocatalytic activity under indoor illumination, coating adhesion after repeated use, and the preservation of the original textile hand feel [129].

Zr-MOF-based textiles can also be engineered as multifunctional protective materials. Cheung and coworkers deposited UiO-66-NH_2_ onto fiber substrates. Subsequent bleaching converted amino groups on organic linkers into reactive N-chlorine active sites. The resulting composite achieved a 7-log reduction against *Escherichia coli* and *Staphylococcus aureus* within five minutes, together with a 5-log reduction in SARS-CoV-2 after 15 min of contact. It also rapidly degraded sulfur mustard simulants. This performance demonstrates the textile platform’s capacity for combined protection against biological and chemical hazards. Recent studies have shifted attention from basic bactericidal efficacy toward bacterial capture, service durability, and multimodal sterilization mechanisms [130]. Gao’s research group anchored silver nanoparticles onto a Zr-MOF and grafted the composite onto cotton fabric modified with quaternized carboxymethyl chitosan (Q-CF). The resulting functional cotton textile combined bacterial capture with dual-mode antibacterial activity. Its antibacterial ratio remained above 85% after repeated washing cycles, mechanical abrasion, immersion in artificial sweat, and long-term storage [95]. Rezaee et al. proposed an optimized strategy for the in situ growth of Zr-MOFs on cotton fabrics. This approach avoided harsh treatment conditions that may damage cellulose structures. The modified textiles exhibited improved hydrophilicity and mechanical properties while maintaining satisfactory washing resistance. In this system, antibacterial activity was readily achieved by loading plant extracts. The results show that Zr-MOF coatings can serve as direct antibacterial components and as stable, long-term carriers for diverse bioactive agents [142].

### 4.3. Food Preservation

Microbial contamination is a widespread problem in the food-processing sector. Bacterial contamination and unfavorable microenvironments within packages, particularly high humidity caused by condensation, accelerate quality deterioration during food storage. Accordingly, active packaging materials should suppress conditions that favor microbial growth and control the sustained release of antibacterial or antioxidant substances. With chemically stable porous frameworks, Zr-MOF fillers can be incorporated into polymer substrates. They can serve as humidity-regulating components and reservoirs for the encapsulation and gradual release of functional molecules, providing new approaches to food safety and quality preservation. Researchers synthesized quantum dot-modified metal–organic framework composite materials (QDs@UIO-66) and constructed visible light-responsive antibacterial packaging materials (quaff) using isolated whey protein amyloid fibrils as the substrate. Under visible light irradiation, quaff achieved an antibacterial rate of 99.87% against Escherichia coli. In postharvest tomato preservation validation, quaff delayed the decline in tomato quality and extended its shelf life to 20 days [143]. Man and coworkers fabricated an iron-zirconium bimetallic metal–organic framework-801 (Fe-MOF-801) packaging material, namely, an iron-zirconium bimetallic-MOF-derived graphitic carbon framework (FeMGCF), with high moisture-adsorption stability. Iron doping strengthened the interaction between the framework and water molecules, and the calculated hydrophilicity increased by 29.6%. This packaging material extended the shelf life of post-harvest tomatoes by six days and slowed the depletion of lycopene and anthocyanin during storage. The study proposed a noncontact antibacterial strategy: reducing accumulated moisture made the package microenvironment less favorable for microbial proliferation while preserving inherent food quality [144].

For food packaging, antimicrobial action should be distinguished from antioxidant protection, humidity regulation, and other preservation mechanisms. Improvements in shelf life alone do not establish microbial killing; examples lacking measured bacterial endpoints are discussed as preservation or environmental-control strategies, not direct antibacterial evidence.

Zr-MOFs can also address the high volatility of plant-derived antibacterial agents. Huang and coworkers selected UiO-66 and MIL-101 as representative nanoscale MOFs and chemically bonded them to carboxymethyl filter paper (CMFP). This produced the compact and flexible nanoMOFs@CMFP composite. After curcumin was encapsulated inside the composite matrix, its chemical stability was more than 4.9-fold higher than that of free curcumin. The curcumin-loaded nanoMOFs@CMFP composite prolonged the shelf life of pitaya fruit and exhibited strong antioxidant and bactericidal activities. This controlled-release mechanism was further demonstrated in the thymol-loaded UiO-66 system [145]. Zhang’s research group modified zirconium-based UiO-66 with a polyethylene glycol coating and loaded thymol into the modified material. The functional filler was then incorporated into chitosan/poly (vinyl alcohol) (CS/PVA) films for antibacterial applications. Experimental data showed that the composite film strongly inhibited *Escherichia coli*, supporting its use in food-packaging materials [146]. Other studies have also examined the antibacterial properties of thymol. Xu and coworkers co-loaded thymol and carvacrol into UiO-66-NH_2_ and blended the composite into chitosan to fabricate antibacterial packaging films. The resulting food-contact film strongly inhibited *Escherichia coli*, *Staphylococcus aureus*, and Flavobacterium citreum [147]. In addition to plant-derived essential-oil components, Wang’s research team prepared sodium-alginate-based packaging films containing melatonin-carrying UiO-66 nanoparticles for preserving fresh spinach. UiO-66 nanofillers strengthened the mechanical robustness and antioxidant capacity of the membrane, thereby maintaining spinach quality during storage. This finding broadens the application scope of UiO-66. Its function extends from the controlled release of antibacterial essential oils to carriers for delivering antioxidant compounds that slow produce senescence and quality deterioration [148].

Porphyrin-functionalized Zr-MOFs further expand this application area by combining controlled release with light-responsive bactericidal activity. Min and coworkers fabricated electrospun pullulan/PVA nanofibers containing thymol-carrying PCN-224 nanoparticles for antibacterial food packaging. PCN-224 served two functions: a carrier for thymol and a photosensitive component capable of generating singlet oxygen [149]. Yan’s research group incorporated polydopamine-coated PCN-224 into gelatin/carboxymethyl cellulose composite films for pork preservation. Upon light irradiation, the film exhibited combined photodynamic and photothermal antibacterial effects. A nanoparticle loading of 5 wt% optimized mechanical strength, water-barrier properties, and ultraviolet shielding, and improved the storage stability of raw pork [150]. Deng and coworkers immobilized carvacrol within PCN-224 nanoparticles and embedded the resulting nanofiller in polycaprolactone/chitosan nanofiber films. Characterization showed that the embedded nanoparticles enhanced the film’s thermal stability and mechanical performance, while simultaneously inhibiting *Escherichia coli* and *Staphylococcus aureus* [151].

### 4.4. Water Treatment

Water treatment presents two scientifically distinct targets: removal or degradation of chemical pollutants, including residual antibiotics, and inactivation of viable microorganisms. Zr-MOF adsorption studies are valuable for environmental remediation, but adsorption capacity for an antibiotic does not itself demonstrate antibacterial disinfection. For the antibacterial scope of this review, direct microbial inactivation, biofouling control, and disinfection performance are treated separately from pollutant-removal metrics. When both functions occur in one system, they should be reported using their respective endpoints rather than combined into a single antibacterial claim.

For water-disinfection applications, relevant performance variables include microorganism identity, initial concentration, contact or irradiation time, water chemistry, organic matter, regeneration/reuse, and leaching of Zr, linkers, or loaded active components. Laboratory results obtained in simple buffers or model water may not predict performance in real water matrices. Zr-MOF-based photocatalytic or electrochemical systems that simultaneously degrade contaminants and inactivate bacteria are therefore most informative when the two outcomes are quantified independently, and material stability is assessed over repeated use. This distinction also prevents pollutant adsorption/degradation studies from being overinterpreted as evidence of microbial disinfection.

Intact Zr-MOFs, Zr-MOF composites, and MOF-derived materials are chemically distinct classes. Following pyrolysis or extensive transformation, the active phase may no longer be an intact coordination framework; antibacterial mechanisms should therefore be attributed to the characterized final material rather than automatically to the parent MOF.

### 4.5. Electrochemical Disinfection and Antibacterial Interfaces

Electrochemical integration can couple Zr-MOF porosity and interfacial chemistry with electrically or photoelectrochemically driven disinfection. Valenzuela and coworkers deposited Zr-NDC and Zr-NDC-2NH2 on graphite electrodes and reported light-driven inactivation of *S. aureus*, including >2-log reduction under UVA irradiation and complete sterilization under the reported full-spectrum sunlight conditions [152]. Defective UiO-66-containing anode membranes have also combined antibiotic oxidation with bacterial inactivation [153], while UiO-66/bamboo-charcoal/chitosan electrodes achieved capacitive desalination together with 99.99% inactivation of *E. coli* under the reported test conditions [154]. These examples illustrate a useful structure–function link: pore accessibility and interfacial redox behavior can support mass transport and reactive-species generation, but antibacterial comparisons still require matched electrical/irradiation conditions, water matrix, bacterial load, and electrode configuration.

Zr-MOF electrochemical sensing platforms can detect bacteria at very low abundance [155,156], but sensing and disinfection are distinct functions. Detection sensitivity or electrochemiluminescence intensity should therefore not be used as evidence of antibacterial efficacy unless the same platform also demonstrates viable-cell inactivation. Within the present antibacterial framework, sensing systems are discussed only when they are integrated with a verified disinfection function; otherwise, they are treated as adjacent applications rather than antibacterial evidence.

## 5. Conclusions and Future Outlook

Zr-MOFs provide a chemically robust and highly tailorable platform for antibacterial materials, but their performance cannot be reduced to a single material property or a single bactericidal mechanism. The most useful structure–function relationships emerge when nanoscale design variables are connected to measurable intermediate properties and then to biological outcomes. Particle size and surface chemistry influence dispersion and bacterial contact; defects and pore accessibility affect active-site exposure, transport, loading, and release; and linker or guest composition determines whether catalytic, photodynamic, photothermal, or drug-delivery pathways are available. These variables can also generate trade-offs, because increased accessibility or reactivity may be accompanied by lower framework stability, faster leaching, or greater nonspecific cellular exposure [20,30,88,89,90].

Across synthesis routes, the key question is therefore not which method is universally superior, but which route reproducibly produces the particle size, defect population, pore environment, surface state, and morphology required for a specific antibacterial context. Solvothermal synthesis offers broad compositional control, mechanochemical and microwave methods can reduce processing time and solvent demand, electrochemical synthesis is attractive for integrated coatings and electrodes, and sonochemical processing can favor rapid nucleation and small crystallites. Direct quantitative comparison remains difficult because many studies do not report the same structural metrics or antibacterial test conditions. Future studies should report particle-size distributions, surface charge, accessible surface area/pore metrics, defect characterization, framework stability, and batch variability together with antibacterial endpoints.

Mechanistically, Zr-MOF antibacterial systems should be separated into at least four evidence-based categories: (i) host-mediated or intrinsic effects of the Zr-MOF, (ii) Zr-MOF-mediated delivery of antibacterial drugs or natural compounds, (iii) Zr-MOF-supported metals/metal oxides or catalytic active species, and (iv) photoactive or multimodal systems. In many high-performing composites, the dominant bactericidal contribution originates from the loaded species or external stimulus rather than from Zr-MOF alone. Appropriate carrier-only, payload-only, dark/light, and pathway-specific controls are therefore essential. The same distinction is required for planktonic antibacterial activity and antibiofilm activity, because biofilm penetration, extracellular polymeric substances, persister cells, and mature-biofilm architecture introduce additional transport and tolerance barriers.

Application-specific evidence should replace broad statements of universal antibacterial performance. Wound systems require safety, biofilm control, stability in complex biological fluids, and evidence of tissue repair; textiles require coating adhesion, wash/wear durability, and activity under realistic illumination or humidity; food-contact materials require controlled migration, storage-condition stability, and preservation of sensory/quality attributes; and water-disinfection systems require independent quantification of pollutant removal and viable-microorganism inactivation in realistic matrices. Pesticide delivery, pollutant adsorption, and sensing-only studies can demonstrate the versatility of Zr-MOFs but should not be used as primary antibacterial evidence when microbial inactivation is not directly measured.

Biosafety requires similarly application-specific analysis. Low toxicity of soluble zirconium species does not establish the safety of an intact Zr-MOF composite. Potential hazards can arise from the framework, organic linkers, modulators, loaded metals or drugs, residual synthesis reagents, and degradation products, and they depend on dose, exposure route, duration, particle size, and degradation/leaching behavior. Claims of biocompatibility should therefore be tied to the actual formulation and test conditions. For translation, hydrolytic stability, sterilization compatibility, aging, leaching, chronic exposure, biodistribution where relevant, and disposal or recovery of spent materials require systematic evaluation.

Several priorities follow from this analysis. First, defect density and particle size should be measured and reported reproducibly rather than used as qualitative descriptors. Second, standardized antibacterial testing and complete reporting of dose, strain, exposure time, irradiation conditions, medium, and controls are needed to enable cross-study comparison. Third, batch-to-batch variability and scalable synthesis should be evaluated together with antibacterial performance. Finally, the field would benefit from studies that deliberately vary one design parameter at a time, or use well-controlled material series, so that causal structure–function relationships can be distinguished from correlations across unrelated formulations.

The expanded benchmark table is intended to reveal both reported values and gaps, rather than to imply universal quantitative transferability. Particularly important missing information includes MIC/MBC, antibiofilm endpoints, defect quantification, leaching, and matched mammalian-cell viability under antibacterial exposure conditions.

In summary, the central design principle for antibacterial Zr-MOFs is not simply to maximize porosity, defects, loading, or reactivity, but to balance these features against stability, controllability, and safety for the intended application. A design-variable-to-property-to-mechanism-to-outcome framework provides a more rigorous basis for comparing Zr-MOF antibacterial systems and for identifying where current evidence remains incomplete. Progress toward practical use will depend on reproducible nanoscale control, mechanistically supported antibacterial claims, standardized testing, and validation under realistic biological or environmental conditions.

## Figures and Tables

**Table 1:** Comparison of recent reviews and the present review.

Review	Scope	Organizing Principle	Evidence Analysis	Principal Conclusions
Ref. [56]	Zr-MOFs across multiple framework families for drug delivery, antitumor therapy, immunoassays, biosensing, and related biomedical applications.	Synthetic routes and Zr-MOF/linker families, linked to their characteristic biomedical functions.	Summarizes synthetic-route advantages/disadvantages and representative biomedical studies; compares linker-dependent modes of action and relates linker functionality and pore dimensions to delivery or photochemical performance.	Tailor linker chemistry and pore structure to the intended biomedical function; advance green synthesis, stimuli-responsive delivery, and studies of degradation, safety, and treatment mechanisms.
Ref. [57]	UiO-based MOFs for antibacterial, anti-inflammatory, and anticancer therapies, with an outlook on AI-assisted materials development.	Disease/therapeutic categories: bacterial infection, inflammation, and cancer, followed by corresponding treatment mechanisms and platforms.	Compiles representative materials, treatment methods, and effects; uses literature-content classification, keyword-frequency statistics, and experimental-data summaries to map research directions.	UiO platforms offer opportunities for controlled/targeted delivery and multimodal therapy; translation requires improved scalability, cost control, in vivo safety, and regulatory readiness, with AI proposed to support optimization.
Ref. [58]	UiO-series antimicrobial materials and delivery systems spanning wound care, environmental disinfection, food preservation, protective coatings, and emerging applications such as microbial fuel cells, agriculture, and oral therapy.	Antimicrobial pathways—controlled release, photocatalytic/photothermal action, and synergistic anti-resistance strategies—followed by application domains.	Synthesizes representative material designs and reported mechanism/performance findings; discusses biosafety gaps, limited in vitro/strain coverage, real-world applicability, and the lack of standardized benchmarking.	Develop precision-targeted, responsive antimicrobial systems while strengthening long-term biosafety evaluation, green/scalable preparation, and standardized assessment for practical translation.
Present review	Antibacterial and antibiofilm Zr-MOF nanomaterials, including UiO and porphyrinic systems, considered across wound care, textiles, food packaging, water disinfection, and electrochemical platforms.	Nanoscale design variable → measurable material property → antibacterial mechanism → biological/application outcome; emphasizes particle size, defects, pore accessibility, surface chemistry, and hybridization.	Critical narrative comparison of material descriptors and antibacterial endpoints; separates host, payload, and stimulus contributions, considers mechanistic support and controls, and interprets results against strain, dose, exposure, irradiation, and test conditions rather than treating heterogeneous studies as directly comparable.	Balance accessibility/reactivity against stability, leaching, and safety instead of maximizing individual properties; prioritize reproducible characterization, matched controls, standardized testing, and controlled material series to distinguish causal structure–function relationships from correlations.

**Table 2:** Representative quantitative antibacterial benchmarks for 12 Zr-MOF-based systems. Full experimental descriptors and NR entries are provided in Supplementary Table S1.

Material/Ref.	Active Design/Loading	Size	BET/Porosity	Defect Descriptor	Zeta Potential	Organism/Exposure	Antibacterial/MIC–MBC/Biofilm	Cytotoxicity
UiO-66-NH_2_–Ag [94]	Ag NPs anchored on UiO-66-NH2; POD-like activity + pH-responsive Ag^+^ release; Ag = 4.396 wt% by ICP-MS	~60 nm composite; Ag NPs ~5 nm	Parent UiO-66-NH_2_: 473 m^2^/g; pore size ~1.5 nm; composite BET NR	NR	+8.06 ± 0.16 mV; parent UiO-66-NH_2_: −5.50 ± 0.47 mV	Amp^r^ *E. coli*; *B. subtilis*; 1 × 10^5^ CFU/mL; material 3–100 μg/mL; 37 °C, 4 h; no external light	At 6 μg/mL, Amp^r^ E. coli survival ~2.2%; at 12 μg/mL, >99.99% killing of both strains; MIC/MBC: NR; biofilm: NR	HUVEC and U87; low/no obvious cytotoxicity at 0–250 μg/mL, 24 h
Zr-TCPP@PDA@FP [109]	Porphyrinic Zr-MOF/PDA paper membrane; NR	NR	NR	NR	NR	*E. coli*; *S. aureus*; visible light/photothermal exposure; exact dose/time NR	>99% inactivation (both); MIC/MBC: NR; visible light 120 min: >99% for *E. coli* and *S. aureus*; biofilm: NR	NR
PB@MOF [112]	Prussian-blue core/porphyrinic Zr-MOF shell; PTT + PDT; ηPT ~29.9%; NA; porphyrin is framework component	PB core ~260 nm; PB@MOF ~300 nm; shell ~20 nm	362.3 m^2^/g; pores ~1.47, 1.61, 1.85 nm; mesopore ~2.64 nm	NR	NR	*S. aureus*; *E. coli*; 100 μg/mL; simultaneous 808 nm + 660 nm irradiation; 10 min	99.31% killing of *S. aureus*; 98.68% killing of *E. coli*; MIC/MBC: NR; biofilm: NR	IH-3T3: ~83% viability at 200 μg/mL after 1 d in dark; dual-light exposure increased cytotoxicity
TiO_2_(2)@ZBPU [118]	TiO_2_/UiO-67 heterojunction; nominal Ti level = 2 mol% formulation	NR	d-ZBPU: 1789 m^2^/g; TiO_2_(2)@ZBPU: 1114 m^2^/g	Missing-linker defects (~1.70 linkers/Zr6 cluster in d-ZBPU); oxygen vacancies (qualitative)	NR	*E. coli*; *S. aureus*; LED irradiation; 30 min	Complete; >90% inactivation; MIC/MBC: NR; biofilm: NR	NR
M-TG dual-ligand Zr-MOF [120]	Porphyrin + ammonium glycyrrhizinate; NR	NR	NR	NR	NR	*E. coli*; *S. aureus*; *B. subtilis*; bacterial suspension: 10^7^ CFU/mL; 900 μL suspension + 100 μL material dispersion (1.5 mg/mL stock; 0.15 mg/mL final); 37 °C, 2 h in dark; LED: 48 W/m^2^, 2 h	Up to 99.999% sterilization; MIC/MBC: NR; biofilm: NR	HUVEC: >85% viability @ 200 μg mL^−1^
NU-1012/cotton [129]	Ti-modified NU-1000; ROS photocatalysis; ~4 Ti atoms per Zr_6_ node in powder	NR	NU-1012 powder: ~954 m^2^/g; pore volume ~0.577 cm^3^/g	Ti incorporated into Zr_6_ node; not a vacancy defect	NR	*E. coli*; *S. epidermidis*; T7 bacteriophage; simulated daylight; ≤1 h	Up to 7-log reduction within 1 h; MIC/MBC: NR; simulated daylight 60 min: 7-log reduction for *E. coli*, S. epidermidis, T7 phage; biofilm: NR	NR
N-chlorinated UiO-66-NH_2_/fiber [130]	Regenerable N–Cl active sites on UiO-66-NH_2_ textile; active chlorine on amine linker; exact tested-batch loading NR	NR	NR	NR	NR	*E. coli*; *S. aureus*; SARS-CoV-2 (antiviral test); direct-contact antibacterial assay (≤5 min); antiviral assay (15 min)	Up to 7-log reduction within 5 min; AATCC-100 test; ≥0.20 wt% Cl: 7-log kill *E. coli*, *S. aureus* within 0.5 min; 3–5 log SARS-CoV-2 reduction in 15 min; MIC/MBC: NR; biofilm: NR	NR
Ag@MOF-545/QCM-cotton [95]	AgNPs + photoactive MOF-545; NR	Ag NPs: 3.6 nm	MOF-545: 1875.80 m^2^/g; Ag@MOF-545: 1009.89 m^2^/g	NR	NR	*E. coli*; *S. aureus*; MRSA; visible light irradiation (10 min; irradiance NR); dark control; repeated washing, abrasion, sweat and storage tests	Initial light-driven killing very high; >85% antibacterial activity retained after durability testing; dark: ~48.6% efficiency; visible light 10 min: >99.9% for *E. coli*, *S. aureus*, MRSA; synergy Ag^+^ + 1O_2_; MIC/MBC: NR; biofilm: NR; bacterial capture evaluated, but no standardized mature-biofilm endpoint	NR
Pt-NP/UiO-66 [125]	Pt nanoparticles immobilized in UiO-66; catalytic ROS generation and biofilm disruption; Pt = 2 wt%, ICP-MS	108 ± 13 nm (composite); Pt NPs: 4 ± 1 nm	UiO-66: 897 m^2^/g; Pt-NP/UiO-66: 873 m^2^/g	NR; no quantitative missing-linker/vacancy analysis	UiO-66: +24.9 ± 3.8 mV; Pt-NP/UiO-66: +29.8 ± 6.1 mV; Pt NP: +0.3 ± 0.2 mV	*S. aureus* Xen36; Mature *S. aureus* Xen36; *S. aureus* Xen36 biofilms matured for 24 h; 24 h treatment with Pt-NP/UiO-66 (up to 800 μg/mL in the biofilm-eradication assay); 400 μg/mL used in macrophage-assisted experiments	Concentration-dependent biofilm disruption and bacterial reduction; effect approached a plateau at approximately 400 μg/mL; MIC/MBC: NR; biofilm: 24 h mature *S. aureus* Xen36 biofilm; endpoints included biofilm thickness and volumetric bacterial density (CFU/biofilm volume). The effect reached a plateau at approximately 400 μg/mL	NIH-3T3 fibroblasts and J774A.1 macrophages; no obvious adverse effect after 24 h up to 600 μg/mL; no significant hemolysis
Au NCs@PCN [126]	Au nanoclusters grown on porphyrinic Zr-MOF (PCN-224); photocatalytic/nanozyme antibacterial platform; NR	~190 nm (SEM/TEM); 196 nm (DLS); Au NCs: ~5–8 nm	NR	NR; Au surface modification, not quantified framework defects	NR	MRSA; ampicillin-resistant *E. coli*; NIR laser irradiation; exact concentration, irradiance, and exposure duration NR (pending verification from full text/SI)	Antibacterial efficiencies of approximately 95.3% (MRSA) and 90.6% (ampicillin-resistant *E. coli*); MIC/MBC: NR; biofilm: MRSA biofilm biomass measured by crystal-violet staining (OD590); biofilm disruption observed	HUVEC and HaCaT cells; approximately 90% viability at 200 μg/mL after 24 h
MOF-525/MOF-545/PCN-223/PCN-22NR4 [127]	MOF-525 (ftw); MOF-545 (csq); PCN-223 (shp); PCN-224 (she). Topology-dependent ^1^O_2_ generation and photodynamic antibacterial activity	MOF-525: 180 × 180 nm; MOF-545: 260 × 40 nm; PCN-223: 230 × 100 nm; PCN-224: ~180 nm	BET: 1687/1079/634/1789 m^2^/g, respectively; pore sizes: 2.0/1.0 and 3.6/1.1/1.9 nm	NR; topology investigated rather than vacancy/missing-linker defects	NR	*E. coli*; *S. aureus*; 0.1 mg/mL MOF; white LED irradiation (4 mW/cm^2^, 20 min)	MOF-545 exhibited the highest antibacterial activity; residual bacterial viability ~0.3% (*E. coli*) and ~0.2% (*S. aureus*); MIC/MBC: MOF-545 MBC: 105 μg/mL (*E. coli*); 100 μg/mL (*S. aureus*); MIC NR; biofilm: NR	NR
Se-MOF [128]	Benzoselenadiazole-containing mixed-linker Zr-MOF (UiO-68 topology); enhanced singlet oxygen generation for photodynamic antibacterial activity; SeTPDC: nominal 20 mol% linker feed; final incorporation NR	~1.2 μm	2882 m^2^/g; average pore size ~2 nm	NR; mixed-linker incorporation rather than quantified structural defects	NR	*S. aureus*; Se-MOF: 50 μg/mL; 450 nm irradiation, 3 mW/cm^2^, 20 min × 3; biofilm evaluation at 6, 12, and 24 h	Light-triggered bacterial inactivation and suppression of biofilm formation; MIC/MBC: NR; biofilm: crystal-violet staining and OD590 biofilm biomass at 6, 12, and 24 h	NR

Note: NR = not reported or not verified in the supplied evidence; experimental conditions and non-equivalent endpoints should not be compared directly. Full 15-column client dataset is retained in Table S1.

## Data Availability

No new data were created or analyzed in this study. Data sharing is not applicable to this article.

## Supplementary Materials

The following supporting information can be downloaded at: https://www.mdpi.com/article/10.3390/nano16201314/s1, Table S1: Complete Table of Representative Quantitative Antibacterial Benchmarks for 12 Zr-MOF-Based Systems.
