# Supplementary material for: Zirconium-Based Metal–Organic Framework Nanomaterials for Antibacterial Applications: Nanoscale Design and Structure–Function Relationships
Source: Nanomaterials (Basel). 2026 Oct 18;16(20):1314. doi: 10.3390/nano16201314 (PMC13707992; doi:10.3390/nano16201314)
Supplement: Supplementary file 1 [file nanomaterials-16-01314-s001.zip › nanomaterials-4600530-supplementary.pdf]

Supplementary Table S1

Complete Table of Representative Quantitative Antibacterial Benchmarks for 12 Zr-MOF-Based Systems.

Material	Active design / structure	Particle size	Porosity / BET	Defect descriptor	Zeta potential	Active-species loading	Organism	Exposure conditions	Antibacterial outcome	MIC / MBC	Antibiofilm endpoint	Model / durability	Cytotoxicity	Ref.
UiO-66-NH$_2$–Ag	Ag NPs anchored on UiO-66-NH$_2$; POD-like activity + pH-responsive Ag$^+$ release	~60 nm composite; Ag NPs ~5 nm	Parent UiO-66-NH$_2$: 473 m^2/g; pore size ~1.5 nm; composite BET NR	NR	+8.06 ± 0.16 mV; parent UiO-66-NH$_2$: -5.50 ± 0.47 mV	Ag = 4.396 wt% by ICP-MS	Amp^R E. coli; B. subtilis	1×10^5 CFU/mL; material 3–100 μg/mL; 37 °C, 4 h; no external light	At 6 μg/mL, Amp^R E. coli survival ~2.2%; at 12 μg/mL, >99.99% killing of both strains	NR	NR	In vitro + infected-wound mouse model; Ag$^+$ release followed up to 10 d	HUVEC and U87; low/no obvious cytotoxicity at 0–250 μg/mL, 24 h	[94]
Zr-TCPP@PDA@FP	Porphyrinic Zr-MOF/PDA paper membrane	NR	NR	NR	NR	NR	E. coli; S. aureus	Visible-light /photothermal exposure; exact dose/time NR	>99% inactivation (both)	NR	NR	Wastewater-treatment membrane; durability NR	NR	[109]
PB@MOF	Prussian-blue core / porphyrinic Zr-MOF shell; PTT + PDT; ηPT ~29.9%	PB core ~260 nm; PB@MOF ~300 nm; shell ~20 nm	362.3 m^2/g; pores ~1.47, 1.61, 1.85 nm; mesopore ~2.64 nm	NR	NR	NA; porphyrin is framework component	S. aureus; E. coli	100 μg/mL; simultaneous 808 nm + 660 nm irradiation; 10 min	99.31% killing of S. aureus; 98.68% killing of E. coli	NR	NR	In vitro + infected-wound model	NIH-3T3: ~83% viability at 200 μg/mL after 1 d in dark; dual-light exposure increased cytotoxicity	[112]
TiO_2(2)@ZBPU	TiO_2/UiO-67 heterojunction	NR	d-ZBPU: 1789 m^2/g; TiO_2(2)@ZBPU: 1114 m^2/g	Missing-linker defects (~1.70 linkers/Zr_6 cluster in d-ZBPU); oxygen vacancies (qualitative)	NR	nominal Ti level = 2 mol% formulation	E. coli; S. aureus	LED irradiation; 30 min	Complete inactivation of E. coli; >90% inactivation of S. aureus	NR	NR	Five reuse cycles reported	NR	[118]
M-TG dual-ligand Zr-MOF	Porphyrin + ammonium glycyrrhizinate	NR	NR	NR	NR	NR	E. coli; S. aureus; B. subtilis	Bacterial suspension: 10^7 CFU/mL; 900 μL suspension + 100 μL material dispersion (1.5 mg/mL stock; 0.15 mg/mL final); 37 °C, 2 h in dark; LED: 48 W/m², 2 h	Up to 99.999% sterilization; growth curves, colony counts and inhibition-zone assays	NR	NR	In vitro bacterial growth and killing assays; durability NR	HUVEC: >85% viability @ 200 μg/mL	[120]
NU-1012/cotton	Ti-modified NU-1000; ROS photocatalysis	NR	NU-1012 powder: ~954 m^2/g; pore volume ~0.577 cm^3/g	Ti incorporated into Zr_6 node; not a vacancy defect	NR	~4 Ti atoms per Zr_6 node in powder	E. coli; S. epidermidis; T7 bacteriophage	Simulated daylight; ≤1 h	Up to 7-log reduction within 1 h	NR	NR	Textile; saline/light aging studied	NR	[129]
N-chlorinated UiO-66-NH$_2$/fiber	Regenerable N–Cl active sites on UiO-66-NH$_2$ textile	NR	NR	NR	NR	Active chlorine on amine linker; exact tested-batch loading NR	E. coli; S. aureus; SARS-CoV-2 (antiviral test)	Direct-contact antibacterial assay (≤5 min); antiviral assay (15 min)	Antibacterial: up to 7-log reduction of E. coli and S. aureus within 5 min. Antiviral: 5-log reduction of SARS-CoV-2 within 15 min	NR	NR	Rechargeable/regenerable textile	NR	[130]
Ag@MOF-545/QCM-cotton	AgNPs + photoactive MOF-545	Ag NPs: 3.6 nm	MOF-545: 1875.80 m^2/g; Ag@MOF-545: 1009.89 m^2/g	NR	NR	NR	E. coli; S. aureus; MRSA	Visible-light irradiation (10 min; irradiance NR); dark control; repeated washing, abrasion, sweat and storage tests	Dark: ~48.6% antibacterial efficiency; visible light: >99.9% against E. coli, S. aureus and MRSA; >85% activity retained after durability tests; $Ag^+/^1O_2$ synergy	NR	NR; bacterial capture evaluated, but no standardized mature-biofilm endpoint	Textile durability	NR	[95]

**Material**	**Active design / structure**	**Particle size**	**Porosity / BET**	**Defect descriptor**	**Zeta potential**	**Active-species loading**	**Organism**	**Exposure conditions**	**Antibacterial outcome**	**MIC / MBC**	**Antibiofilm endpoint**	**Model / durability**	**Cytotoxicity**	**Ref.**
Pt-NP/UiO-66	Pt nanoparticles immobilized in UiO-66; catalytic ROS generation and biofilm disruption	108 ± 13 nm (composite); Pt NPs: 4 ± 1 nm	UiO-66: 897 m^2/g; Pt-NP/UiO-66: 873 m^2/g	NR; no quantitative missing-linker/vacancy analysis	UiO-66: +24.9 ± 3.8 mV; Pt-NP/UiO-66 : +29.8 ± 6.1 mV; Pt NP : +0.3 ± 0.2 mV	Pt = 2 wt%, ICP-MS	S. aureus Xen36	S. aureus Xen36 biofilms matured for 24 h; 24 h treatment with Pt-NP/UiO-66 (up to 800 µg/mL in the biofilm-eradication assay); 400 µg/mL used in macrophage-assisted experiments	Concentration-dependent biofilm dispersal, approaching a plateau at 400 µg/mL; enhanced bacterial clearance in the presence of macrophages	NR	Biofilm thickness, volumetric bacterial density, LIVE/DEAD staining, and viable bacterial counts (CFU)	In vitro mature biofilm and in vivo infected-wound model; long-term durability NR	NIH-3T3 fibroblasts and J774A.1 macrophages; no obvious adverse effect after 24 h up to 600 µg/mL; no significant hemolysis	[125]
Au NCs@PCN	Au nanoclusters grown on porphyrinic Zr-MOF (PCN-224); photocatalytic/nanozyme antibacterial platform	~190 nm (SEM/TEM); 196 nm (DLS); Au NCs: ~5–8 nm	NR	NR; Au surface modification, not quantified framework defects	NR	NR	MRSA; ampicillin-resistant E. coli	NIR laser irradiation; exact concentration, irradiance, and exposure duration NR (pending verification from full text/SI)	95.3% inhibition of MRSA and 90.6% inhibition of ampicillin-resistant E. coli; photothermal temperature up to 56.2 °C	NR	MRSA biofilm biomass measured by crystal-violet staining (OD590); biofilm disruption observed	Infected diabetic-rat wound model; residual wound area decreased to 2.7% by day 21; long-term material durability NR	HUVEC and HaCaT cells; approximately 90% viability at 200 µg/mL after 24 h	[126]
MOF-525 /MOF-545/PCN-223/PCN-224	MOF-525 (ftw); MOF-545 (csq); PCN-223 (shp); PCN-224 (she). Topology-dependent $^1O_2$ generation and photodynamic antibacterial activity	MOF-525: 180 × 180 nm; MOF-545: 260 × 40 nm; PCN-223: 230 × 100 nm; PCN-224: ~180 nm	BET: 1687 / 1079 / 634 / 1789 m^2/g, respectively; pore sizes: 2.0 / 1.0 & 3.6 / 1.1 / 1.9 nm	NR; topology investigated rather than vacancy/missing-linker defects	NR	NA; TCPP photosensitizer incorporated as framework linker	E. coli; S. aureus	0.1 mg/mL MOF; white LED irradiation (4 mW/cm^2, 20 min)	MOF-545 exhibited the highest antibacterial activity; residual bacterial viability ~0.3% (E. coli) and ~0.2% (S. aureus) Activity ranking: MOF-545 > MOF-525 > PCN-224 > PCN-223.	MOF-545 MBC: 105 µg/mL (E. coli); 100 µg/mL (S. aureus); MIC NR	NR	In vitro planktonic bacterial assays; durability NR	NR	[127]
Se-MOF	Benzoselenadiazole-containing mixed-linker Zr-MOF (UiO-68 topology); enhanced singlet oxygen generation for photodynamic antibacterial activity	~1.2 µm	2882 m^2/g; average pore size ~2 nm	NR; mixed-linker incorporation rather than quantified structural defects	NR	SeTPDC: nominal 20 mol% linker feed; final incorporation NR	S. aureus	Antibacterial assay: initial bacterial density 10^5 CFU/mL; 450 nm LED, 3 mW/cm^2, 20 min per irradiation, repeated three times; material concentration varied. Biofilm assay: 10^7 CFU/mL, 50 µg/mL Se-MOF; same irradiation protocol; incubation for 6, 12, or 24 h	Light-triggered bacterial inactivation and suppression of biofilm formation	NR	Inhibition of S. aureus biofilm formation, quantified by crystal-violet staining (OD_{590}) after 6, 12, and 24 h	In vitro bacterial and biofilm assays; durability NR	NR	[128]
